# A novel dataset for encrypted virtual private network traffic analysis

**DOI:** 10.1016/j.dib.2023.108945

**Published:** 2023-02-01

**Authors:** Mohamed Naas, Jan Fesl

**Affiliations:** a Department of Informatics, University of South Bohemia, Faculty of Science, Czechia; b Department of Computer Systems, Czech Technical University in Prague, Faculty of Information Technology, Czechia

**Keywords:** Machine Learning, IP flow, IPFIX, Network traffic, SSTP, OpenVPN, Wireguard

## Abstract

Encryption of network traffic should guarantee anonymity and prevent potential interception of information. Encrypted virtual private networks (VPNs) are designed to create special data tunnels that allow reliable transmission between networks and/or end users. However, as has been shown in a number of scientific papers, encryption alone may not be sufficient to secure data transmissions in the sense that certain information may be exposed. Our team has constructed a large dataset that contains generated encrypted network traffic data. This dataset contains a general network traffic model consisting of different types of network traffic such as web, emailing, video conferencing, video streaming, and terminal services. For the same network traffic model, data are measured for different scenarios, i.e., for data traffic through different types of VPNs and without VPNs. Additionally, the dataset contains the initial handshake of the VPN connections. The dataset can be used by various data scientists dealing with the classification of encrypted network traffic and encrypted VPNs.

**Specifications Table**

**Table:** 

Subject	Computer Networks and Communications

Specific subject area	Encrypted Private Virtual Networks and their classification.
Type of data	Structured
How the data were acquired	Data was obtained by simulating real-world traffic through network traffic probes, stripped of redundant information and organized into flows in a sense of context. The packet resolution at the time level is in the order of microseconds. The data was captured from Mikrotik RouterOS routers using the open-source software pmacct. The data was exported in the IPFIX [1] format into Apache Kafka, preprocessed using our own solution *ipFlowDetector* and finally exported to JSON files.
Data format	Raw
Description of data collection	The data was measured in our network laboratory by specific conditions and scenarios described in Section 2. The data was not rearranged but was filtered from insufficient traffic flows.
Data source location	• Department of Informatics, Faculty of Science, University of South Bohemia. • Branišovská 31a, České Budějovice, Czech Republic, 37,001. • GPS Coordinates: 48° 58′ 28.09″ N, 14° 28′ 27.62″ E
Data accessibility	Repository name: Zenodo Data identification number: 10.5281/zenodo.7301756 Direct URL to data: https://zenodo.org/record/7301756

## Value of the Data

• Researchers can utilize the data to investigate patterns in the behavior of different VPN protocols and traffic types, and use this information to create machine learning models that can detect VPN usage and classify VPN and traffic types.
• The proposed dataset can serve as a benchmark for researchers and developers studying VPN and traffic patterns. By utilizing machine learning and other algorithms, researchers can investigate patterns in VPN behavior and use this information to improve network Quality of Service (QoS) [2,3], investigate the privacy and security risks of each VPN type [4], or potentially develop VPN architectures that can bypass ISP and government restrictions.
• The dataset includes a diverse range of VPN protocols, including L2TP [5], L2TP-IPSEC [5], PPTP [6], SSTP [7], WireGuard [8], and OpenVPN [9]. Additionally, the dataset includes initial handshake flows for each VPN type, providing valuable information for further analysis. To the best of our knowledge, this is the first dataset to include multiple types of VPN flows beyond OpenVPN.

## Objective

The dataset aims to allow researchers to study and compare the different VPN types and internet traffic. We included multiple VPN types that are less studied in the literature compared to OpenVPN, making them more visible and accessible to researchers. And since many new web services emerged in recent years, we used new and more up-to-date versions of services and websites compared to similar datasets.

The most comparable dataset ISCXVPN2016 [10] contains a limited VPN variety and outdated traffic content (given that the content used to generate the data is older than 6 years). While newer datasets such as [4] and [11] only use OpenVPN and the data is not open-source for researchers to use. Making our dataset an important asset for scientists.

## 1 Data Description

The dataset consists of labeled network traffic. The traffic is either a VPN traffic or a non-VPN traffic. The VPN traffic is generated via a set of different VPN types:

• PPTP: Point-to-Point Tunneling Protocol built by Microsoft, it operated in Layer 2 of the OSI Model [12], with not sufficient encryption level [13].
• L2TP: Layer 2 Tunneling Protocol (in the OSI Model) without data encryption or strong authentication.
• L2TP-IPSEC: Layer 2 Tunneling Protocol, encrypted using IPSEC protocol (NAT-Traversal mode).
• SSTP: Secure Socket Tunneling Protocol, based on HTTPS and operates at the application layer of the OSI model.
• WireGuard: Modern and open-source VPN protocol that operates on layer 7, utilizes state of art cryptography techniques, and uses UDP as its transport protocol.
• OpenVPN: Modern, popular, and open-source protocol that operated on layer 7. It is used primarily for end-user connections.

We divided the generated traffic into seven types of traffic:

• Non-streaming: HTTP/HTTPS traffic from websites that do not contain streaming content such as videos and audios. Example websites are www.google.com or www.github.com.
• Streaming: HTTP/HTTPS traffic from websites that contain streaming content like Youtube and Twitch.
• Email: Traffic generated from delivering emails.
• VoIP: Traffic generated from videoconferencing services such as Google Meet.
• SSH: Traffic generated from connecting to remote servers using Secure Shell protocol.

In addition to the flows of the generated traffic, we also included the first flows of each VPN's initial connection.

**Fig. 1:**
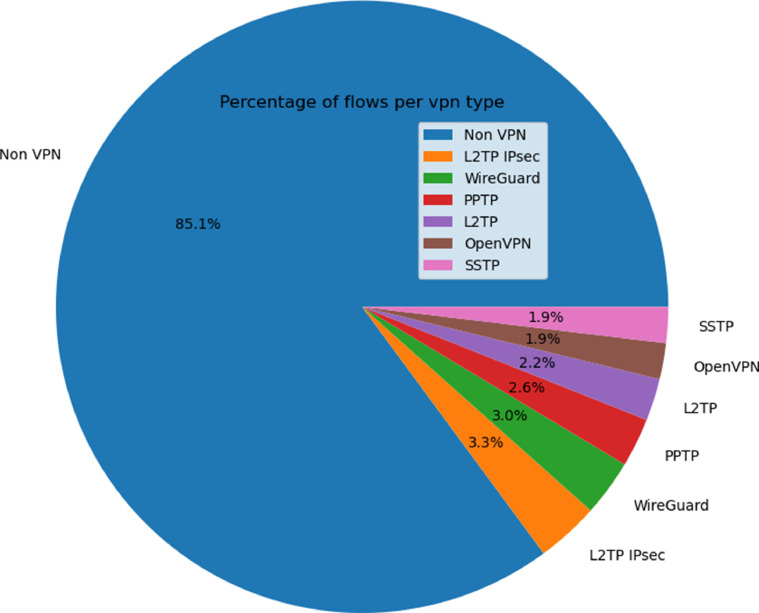
Pie chart of the distribution of the flows for each VPN type. Each slice of the pie chart represents the percentage of flows for a particular VPN type on the total dataset flows.

**Fig. 2:**
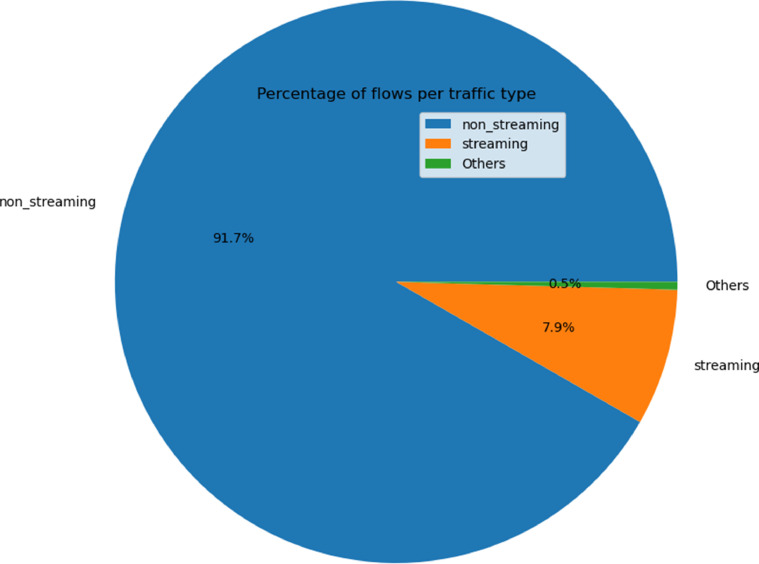
Pie chart of the distribution of the flows for each traffic type. Each slice of the pie chart represents the percentage of flows for a particular traffic type on the total dataset flows.

Table 1 demonstrates that the dataset contains a substantial number of flows and is varied among different types of VPNs. Fig. 1 illustrates the distribution of VPN flows in the dataset, with each slice of the pie chart representing the percentage of flows for a particular VPN type on the total dataset flows. And similarly for traffic types in Fig. 2.

**Table 1:** The number of flows and the size of the dataset for each VPN traffic type (without counting the initial flows).

	Vpn type	Size	Number of flows
Non-VPN traffic		4.3GB	50,191
VPN traffic	L2TP	1.7GB	1314
	L2TP-IPSEC	2.5GB	1955
	PPTP	2.3GB	1521
	SSTP	2.8GB	1118
	WireGuard	2.6GB	1758
	OpenVPN	2.3GB	1120
	Total	14.2GB	6861
Overall Total		18GB	58,977

**Fig. 3:**
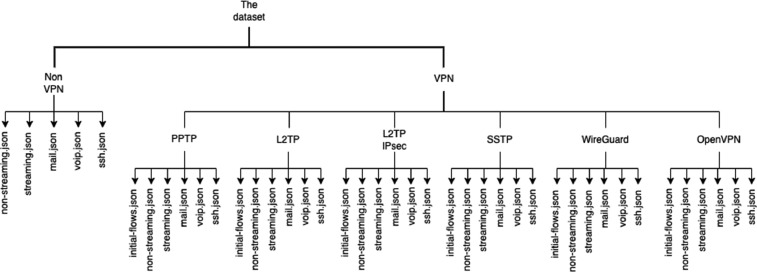
The structure of folders and files in the dataset.

**Fig. 4:**
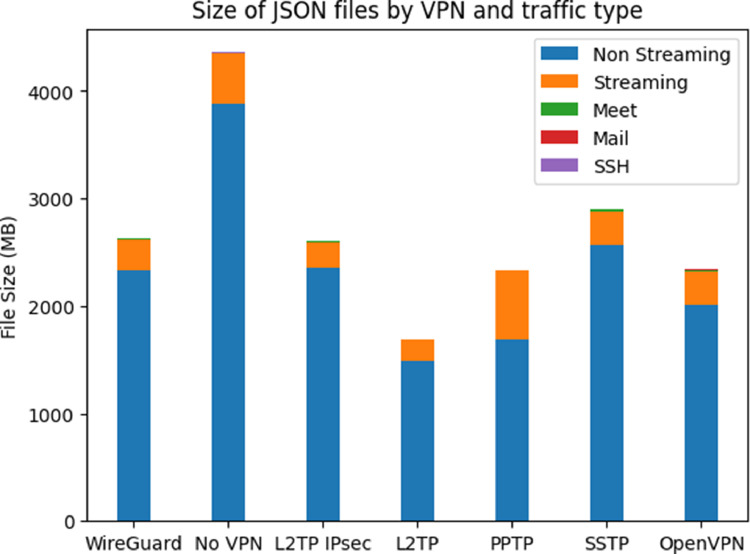
The size of the dataset by VPN and traffic type.

The dataset is stored in the JSON format which is readable and supported by modern programming languages. The dataset on the top level is split into two folders, the first contains non-VPN flows while the second contains VPN flows. In the last folder, there are six folders for each type of VPN. The non-VPN folder contains five traffic JSON files, while each of the VPN folders has five traffic JSON files plus a JSON file containing the first flows when establishing the VPN connection. Fig. 3 demonstrates how the files are organized in the dataset and Fig. 4 shows the sizes of the dataset by VPN and traffic type.

In each JSON file, there is an array of flows. A flow is represented as an object where its principal information are stored such as protocol name and used ports. The description of the flow object is found in Table 2.

**Table 2:** The description of the flow object attributes.

Attribute Name	Description
ip_proto	Name of the protocol
port_dst	Destination port
port_src	Source port
x_packets	Array of the captured packets in the flow

Inside all flow objects, there is an array of the captured packets during that flow. Each packet is represented as a JSON object. The presence of attributes in packets may differ from one flow protocol to the other. The description of the packet object is in Table 3.

**Table 3:** The description of the packet object attributes.

Attribute Name	Description
bytes	The size of the payload of the packet If the value was positive it means that the packet was in the forward direction. Otherwise, the packet was in the backward direction
timestamp_start	The start timestamp of the captured packet
timestamp_end	The start timestamp of the captured packet
packets	The number of the captured packets during the capturing timestamp
ip_header_len	The length of IP header
tcp_header_len	The length of the TCP header
tcp_ack_number	The TCP acknowledgement number
tcp_flags	The TCP flags
tcp_seq_number	The TCP sequence number

## 2 Experimental Design, Materials and Methods

In this section, we describe the environment used for establishing and collecting VPN and non-VPN and network flows (Section 2.1), then we provide an overview of the data acquisition process (Section 2.2).

### 2.1 The Data Measurement Scheme

**Fig. 5:**
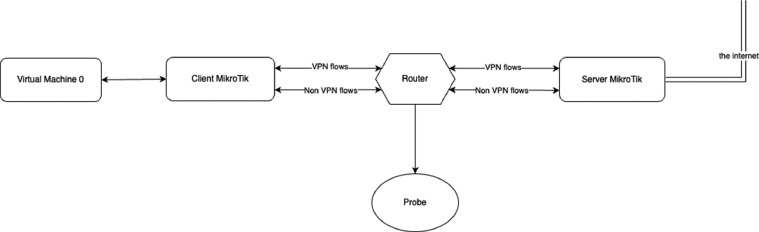
The topology of the network items used for the traffic generation and capturing. All items are described in detail in the list above.

In Fig. 5, we show the environment used for the data acquisition. The scheme consists of five main components used for flow generation, VPN connection establishment, and capturing/filtering the network flows. The detailed overview of the roles and the specifications of each component is as follows:

• *Virtual Machine 0 (VM0):* An Ubuntu 20.04 LTS virtual machine with the purpose of generating web traffic and storing the captured flows. It receives the captured flows from the *Probe* passing by *Client MikroTik,* then saves them. It also receives and sends traffic from and to *Client MikroTik*.
• *Client MikroTik:* A MikroTik RouterOS virtual machine, it plays the role of a client in the VPN mode, and it links the Router and the VM0. The VPN type is set manually in this VM.
• *Server MikroTik:* A MikroTik RouterOS virtual machine, it plays the role of a server in the VPN mode, and it links the Router and the internet. The VPN type is set manually in this VM.
• *Router:* A physical router hosted in the university laboratory. It links the *Client* and the *Server MikroTik* virtual machines and sends the passing packets to the probe.
• *Probe:* A physical computer that captures the mirrored traffic coming from the router, converts the traffic into the IPFIX format, and uploads the IPFIX records to a data storage.

The MikroTik RouterOS already includes the configurations of all of the used VPNs. In the non-VPN setup, we disabled all of the VPN configurations and routed the traffic from *VM0* directly through the *Router*.

The captured traffic from the probe is preprocessed and filtered using *ipFlowDetector*, a program that we made using the C++ programming language for efficiency purposes, then finally we exported the resulting flows into JSON files and stored them in *VM0*. The JSON files are later on anonymized from IP addresses and further filtered from broadcasting flows.

### 2.2 Traffic Generation

In our work, we divided the generated traffic into five types: streaming, non-streaming, mail, VoIP, and SSH (refer to Section 1 for each type description). The choice of this classification and the distribution of each type was mainly based on our intuition because there are few publications on the distribution of traffic types in the real world [14].

To automate the traffic generation process we created shell and python scripts. Each python script contains the automatization of a traffic type. While the shell script contains the order of commands to run *ipFlowDetector* program and the python script between different VPN and traffic types. The details of the automatization of each traffic type are as follows:

• Non-streaming: *Selenium* library and Google Chrome version 104 were used. we collected a list of 1022 website URLs that do not contain streaming content, such websites are Wikipedia and Pinterest. The script opens the websites sequentially, waits for the page to load, stays on the page for a short duration then moves to the next website.
• Streaming: *Selenium* python and Google Chrome version 104 were used. We collected a list of 105 streaming content mostly from Youtube; the rest are from Twitch, SoundCloud, and other streaming services. Similarly to *non-streaming*, the script opens the 105 websites sequentially but stays in them for a longer time.
• VoIP: *Selenium* python and Google Chrome version 104 were used. We used google meet (voice and video), with a simulated camera on the side of *VM0*.
• Mail: Sent multiple emails using *redmail* library and outlook.
• SSH: Connected to a remote terminal and executed a list of commands multiple times using *spur* library.

In the VPN mode, *ipFlowDetector* captured the initial flows of each VPN connection establishment and saved them in *initial-flows.json*. The motivation for including these flows in the dataset is that OpenVPN handshakes have been used as a VPN fingerprinting method [4] and it can be useful for researchers to investigate other VPNs' handshakes. After establishing the VPN connection, we started capturing the flows of the five types of traffic.

## Ethics Statements

Our work does not contain information retrieved from human subjects or based on animal experiments.

## CRediT authorship contribution statement

**Mohamed Naas:** Data curation, Writing – review & editing. **Jan Fesl:** Conceptualization, Methodology, Supervision.

## Declaration of Competing Interest

The authors declare that they have no known competing financial interests or personal relationships that could have appeared to influence the work reported in this paper.

## Data Availability

USBVPN2022 (Original data) (zenodo)
